# Medical, Surgical, and Combined Approaches in Pediatric Hydatid Liver Disease: A Systematic Review

**DOI:** 10.3390/pediatric18020038

**Published:** 2026-03-05

**Authors:** Amani N. Alansari, Marwa Messaoud, Salma Mani, Amine Ksia

**Affiliations:** 1 Department of Pediatric Surgery, Hamad Medical Corporation, Doha 3050, Qatar; 2 Pediatric Surgery Department, Fattouma Bourguiba University Hospital, Monastir 5000, Tunisia; marwa.mesaoud@gmail.com (M.M.); salmamanihs@hotmail.com (S.M.); amineks@yahoo.fr (A.K.); 3 Faculty of Medicine of Monastir, University of Monastir, Monastir 5000, Tunisia; 4 Research Laboratory of Congenital Anomalies and Childhood Cancer LR12SP13, University of Monastir, Monastir 5000, Tunisia

**Keywords:** hydatid liver disease, echinococcosis, pediatric, medical therapy, surgical intervention

## Abstract

**Background:** Hydatid disease poses unique management challenges in pediatric populations due to developing anatomy and growth considerations. This systematic review evaluates the efficacy and safety of medical, surgical, and combination therapies for pediatric hydatid liver disease. **Methods:** A comprehensive search of PubMed, Scopus, Web of Science, and Cochrane Library from inception to January 2025 identified studies investigating treatment outcomes in pediatric hydatid liver disease. Data was synthesized through qualitative analysis of treatment effectiveness, complications, and patient outcomes. **Results:** Fifteen studies were included, comprising controlled trials, cohort studies, and cross-sectional studies. Treatment efficacy correlated significantly with cyst size: small cysts (<5 cm) responded well to albendazole monotherapy (88.3–97.6% success at 6–12 months); medium-sized cysts (5–6 cm) benefited from percutaneous interventions (PAIR) with 97.1% technical success; large cysts (>6 cm) required surgical management. Laparoscopic approaches demonstrated advantages over open surgery, including shorter hospitalization (5.6 ± 2.2 vs. 12.1 ± 1.5 days) and reduced analgesic requirements. Omentoplasty emerged as superior for residual cavity management with fewer complications than tube drainage approaches. **Conclusions:** This review supports personalized treatment algorithms based primarily on cyst characteristics. The findings recommend standardized protocols incorporating cyst size, location, and complexity as key decision points, with expanded access to minimally invasive techniques. Future research should focus on prospective comparative studies with standardized outcome measures.

## 1. Introduction

Hydatid disease, caused by the parasitic tapeworm *Echinococcus*, remains a significant public health challenge in endemic regions, particularly affecting pediatric populations with potentially severe complications [1]. The management of hydatid cysts in children presents unique therapeutic challenges due to the developing anatomy, long-term growth considerations, and the need for less invasive treatment approaches [2,3]. While various treatment modalities exist—ranging from purely medical management to surgical intervention or combination therapy—the optimal treatment strategy for pediatric patients remains a subject of ongoing debate in the medical community [2,4].

The World Health Organization estimates that hydatid disease affects approximately 1 million people globally at any given time, with children comprising 15–20% of cases in endemic regions [5]. The prevalence in pediatric populations is particularly concerning due to the potential for prolonged disease progression and the impact on physical development [6]. The liver (70%) and lungs (20%) represent the most commonly affected organs in children, often requiring carefully tailored therapeutic approaches that balance treatment efficacy with minimizing procedural risks [7,8].

Current treatment modalities encompass three primary approaches: medical therapy using benzimidazoles (primarily albendazole), surgical intervention (ranging from complete cyst excision to minimally invasive techniques), and combination therapy [9]. Each approach carries distinct advantages and limitations. Medical treatment offers a non-invasive option but may require prolonged administration periods and carries the risk of incomplete resolution. Surgical intervention provides immediate cyst removal but involves operative risks and potential complications such as infection, bleeding, and damage to nearby structures [10]. Combination therapy potentially combines the benefits of both approaches while potentially mitigating their individual drawbacks [2,10]. In children, untreated or inadequately treated hydatid disease may result in continued cyst growth, rupture, secondary dissemination, and long-term impairment of organ function and development, emphasizing the clinical importance of determining optimal management strategies [11].

Despite numerous studies examining these treatment modalities, the comparative effectiveness of these approaches in pediatric populations is still lacking. Previous meta-analyses—including adult surgical comparisons of open versus laparoscopic techniques [12] and evaluations of PAIR versus laparoscopic procedures [13]—have largely focused on adult cohorts without providing pediatric-specific effectiveness data, and a recent meta-analysis on the incidence of hydatid disease in children highlights the significant but variable pediatric disease burden [14]. Furthermore, the unique considerations in pediatric cases—including growth potential, organ plasticity, and long-term outcomes—necessitate a specialized analysis focused on this vulnerable population [15].

This systematic review evaluates the efficacy and safety of treatment options for pediatric liver hydatid cysts, with a particular focus on comparing medical therapy, surgical intervention, and combination approaches. Specifically, it examines key outcomes including treatment success, complication rates, and recurrence to address the existing uncertainty regarding optimal management strategies in children. The findings will guide clinical decision-making, particularly in endemic regions, optimizing resource allocation and treatment strategies. This study aims to inform practice and highlight research gaps, contributing to evidence-based protocols for pediatric hydatid liver disease management.

## 2. Materials and Methods

### 2.1. Study Design

This systematic review was conducted following the Preferred Reporting Items for Systematic Reviews and Meta-Analyses (PRISMA) guidelines and Cochrane handbook [16,17,18] (Supplementary Materials). The protocol was registered in PROSPERO (CRD420251247688).

### 2.2. Eligibility Criteria

Studies were included if they met the following criteria: (1) examined pediatric patients with hepatic hydatid disease; (2) reported treatment outcomes of medical and/or surgical interventions; and (3) included retrospective or prospective cohort studies or clinical trials. Exclusion criteria included studies with insufficient data, case reports, case series, or any study with a small sample size (<10), editorials, non-English articles, and studies including mixed adult and pediatric populations unless pediatric data were reported separately.

### 2.3. Information Sources, Search Strategy, and Study Selection

A comprehensive search was conducted across PubMed, Scopus, Web of Science (WoS), and the Cochrane Library from inception to January 2025. The search strategy included a combination of MeSH terms and keywords related to echinococcosis, hydatid disease, liver, and pediatrics s, using explicit Boolean operators, and no filters were applied. All identified records were imported into EndNote for duplicate removal. Two independent reviewers screened the titles and abstracts, with full-text articles assessed against eligibility criteria. Discrepancies were resolved by a third reviewer.

### 2.4. Data Collection and Extraction

A standardized data extraction form was used to collect study characteristics, patient demographics, baseline clinical characteristics, potential confounding factors, treatment protocols (including type, duration, and dosage), and outcomes. Data were independently extracted by two reviewers, and discrepancies were resolved by consensus.

### 2.5. Quality Assessment

The quality of the included studies was assessed using standardized tools based on study design. The Newcastle-Ottawa Scale (NOS) was employed for cohort and cross-sectional studies, which assesses three domains: selection of study groups, comparability of cohorts based on key confounding variables, and assessment of outcomes; each study received a score from 0 to 9, with scores ≥6 considered fair to good quality [19]. Non-randomized controlled trials (RCTs) were evaluated using the Risk of Bias in Non-Randomized Studies of Interventions (ROBINS-I) tool, which evaluates risk of bias across seven domains: confounding, selection of participants, classification of interventions, deviations from intended interventions, missing data, measurement of outcomes, and selection of reported results [20]. RCTs were assessed using the Cochrane Risk of Bias 2 (ROB-2) tool, which considers randomization, deviations from intended interventions, missing outcome data, measurement of outcomes, and selective reporting [21]. Two independent reviewers conducted the assessments, with a third reviewer resolving conflicts.

Quality assessments were used to inform the synthesis by highlighting the risk of bias in individual studies; studies of lower quality were not excluded but were given less weight in the narrative synthesis, and their limitations were explicitly considered when interpreting the results.

### 2.6. Data Synthesis

A qualitative synthesis of the included studies was conducted, emphasizing key aspects such as treatment effectiveness, reported complications, and overall patient outcomes. Due to significant heterogeneity in study designs, interventions, outcome measures, and patient characteristics, a formal meta-analysis could not be performed.

## 3. Results

**Figure 1:**
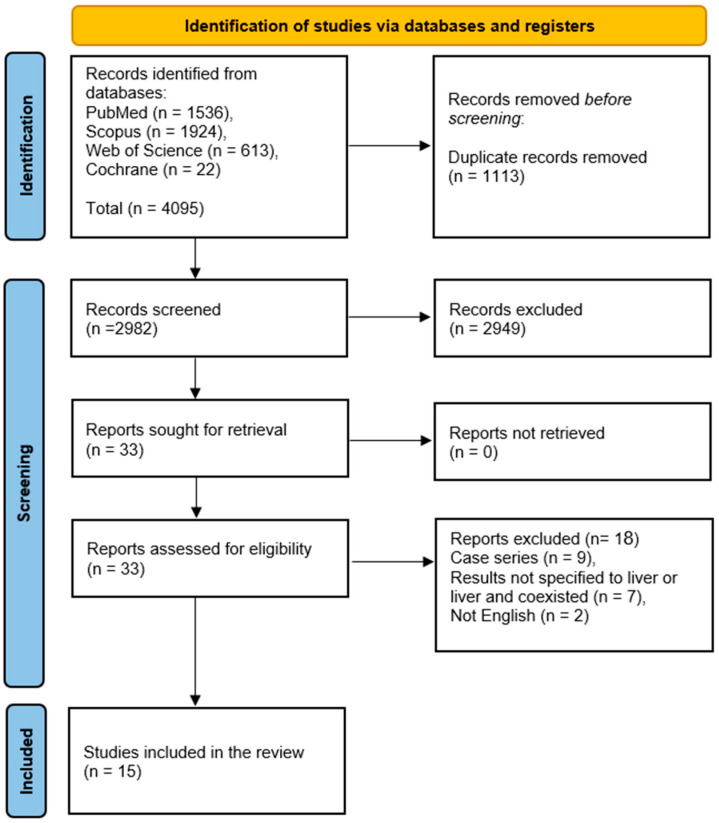
PRISMA flow diagram.

A comprehensive database search across PubMed, Scopus, WoS, and Cochrane identified 4095 records. After removing 1113 duplicates, 2982 records underwent title and abstract screening, with 2949 excluded. A total of 33 reports were sought for retrieval. Following full-text screening of 33 reports, 18 studies were excluded based on eligibility criteria: 9 were case series, 7 did not involve liver hydatid cysts, and 2 were non-English publications. Ultimately, 15 studies were included in this review [6,22,23,24,25,26,27,28,29,30,31,32,33,34,35] (Shown in Figure 1).

### 3.1. Baseline and Summary of the Included Studies

The studies vary in design, including 8 cohort studies, five non-RCTs, one RCT, and one cross-sectional study, with sample sizes ranging from 14 to 214 patients.

The interventions assessed include medical management with albendazole or mebendazole, percutaneous drainage techniques such as PAIR (Puncture, Aspiration, Injection, and Re-aspiration), and various surgical approaches, including laparoscopic and open surgery. Some studies compared different surgical techniques, while others focused on the effectiveness of medical therapy alone.

The diagnostic tools commonly used across studies included ultrasound (US), computed tomography (CT), serological tests such as the indirect hemagglutination test (IHA), and liver function tests. Follow-up periods varied, ranging from months to several years, with primary outcomes including cyst recurrence, treatment success, and post-intervention complications.

The patient demographics varied across studies, with a predominant focus on pediatric populations, ranging from 2 to 17.5 years of age. The median or mean ages reported in the studies varied, with some reporting a mean age of approximately 9–11 years. The male-to-female ratio was relatively balanced in most studies, though some reported a higher prevalence in males. Further details are shown in Table 1.

### 3.2. Quality Assessment

The quality of the included studies was assessed using various tools appropriate to each study design. Cohort studies were evaluated using NOS, with scores ranging from 6 to 9. Studies such as Azizoğlu (2024), Aygün (2020) and Dolanbay (2020) received a score of 6 [6,23,24], indicating fair quality, whereas the other 5 cohort studies scored 7–9, reflecting good quality. Non-RCTs were appraised using the ROBINS-I tool. Studies varied in risk of bias. Minaev et al. (2017) had the lowest risk [27], while Khursheed et al. (2001) exhibited serious concerns [33]. In contrast, Goktay et al. (2005) and Wu et al. (1992) demonstrated a moderate to serious risk of bias [25,35]. The cross-sectional study by Pradhan (2022), assessed using the NOS tool, received a score of 6, indicating fair quality [28,29]. The only RCT, Masood (2022), assessed using the ROB-2 tool, exhibited some concerns, particularly regarding the randomization process [26]. Further details are shown in Table A1, Table A2, Table A3 and Table A4.

### 3.3. Qualitative Synthesis

#### 3.3.1. Diagnostic Approaches and Initial Presentation

Diagnosis of hepatic hydatid disease in pediatric populations consistently employs multimodal approaches across studies. Serological testing demonstrated variable sensitivity, with immunohemagglutination assay (IHA) positivity rates ranging from 60% [31] to 66.1% [23]. Despite confirmed disease, serological negativity was observed in 40% of patients in Sağ’s cohort, highlighting the limitations of serological diagnosis alone. Enzyme-linked immunosorbent assay (ELISA) demonstrated superior sensitivity compared to IHA, with reported rates of 84.4% [25] and 86% [33], suggesting its preferential use where available.

Eosinophilia demonstrated significant variability as a diagnostic marker, with rates of 21% [33], 30.4% [23] and 55% [6], indicating its insufficient reliability as a standalone diagnostic parameter.

Clinical presentation patterns showed remarkable consistency across studies, with abdominal pain representing the predominant symptom, reported in 55.4% [23], 66.4% [32], 67.6% [25], and 77.1% of patients [31]. Wu’s (1992) diagnostic protocol incorporated epidemiological factors, with all 43 children residing in endemic areas and 39 (90.7%) reporting contact with dogs [35]. This epidemiological correlation was similarly documented by Aygün (2020), who reported that 44.6% of patients had documented canine contact history [23].

US emerged as the primary diagnostic imaging modality, with CT reserved for complex cases or diagnostic uncertainty. Incidental diagnoses were identified in 13.1% of cases, indicating that the disease may remain asymptomatic despite the presence of sizable cysts. Goktay (2005) reported that 26.5% of patients were asymptomatic at presentation [25].

#### 3.3.2. Medical Therapy Regimens and Timing

Medical therapy protocols demonstrated significant evolution over time, with albendazole replacing mebendazole as the preferred agent in contemporary practice. This shift is primarily due to albendazole’s superior efficacy in treating hydatid cysts, as it achieves better tissue penetration and has a more favorable pharmacokinetic profile. Furthermore, albendazole is effective at lower doses and has been associated with a reduced risk of recurrence compared to mebendazole [36]. Dosing standardization emerged with albendazole consistently administered at 10–15 mg/kg/day [27,28,29,31], contrasting with historical mebendazole regimens at substantially higher dosages 50–100 mg/kg/day in Wu, 1992 [35]; 100–200 mg/kg/day in Messaritakis, 1991 [34].

Treatment duration and timing protocols varied significantly across studies. Preoperative administration ranged from 1 to 6 weeks, with Sağ (2022) implementing a three-week preoperative course for all surgical candidates [31]. Masood (2022) implemented a higher dose regimen of albendazole at 15 mg/kg/day for a minimum of 4 weeks preoperatively, specifically designed to reduce cyst viability and minimize the risk of intraoperative spillage [26]. Postoperative regimens have evolved considerably over time. Wu (1992) employed a structured postoperative mebendazole protocol of three 28–30 day courses separated by monthly intervals, designed to optimize absorption and minimize hepatotoxicity [35]. Later approaches showed greater heterogeneity, with Ran (2015) reporting shorter courses (14–21 days) [30]. Masood (2022) recently developed an extended cyclical approach with 3-week treatment periods separated by 1-week intervals, representing a shift toward intermittent dosing strategies [26].

Monitoring protocols during medical therapy demonstrated remarkable consistency, with liver function tests performed every 2–4 weeks [22,28,31]. Tolerability concerns were reported across studies. Goktay (2005) documented aminotransferase elevation in 12% of patients at one-month follow-up, necessitating treatment discontinuation. Additionally, 9% of patients exhibited gastric intolerance after 1–3 weeks of treatment [25].

Oral (2012) reported that small cysts (<5 cm) managed with albendazole monotherapy achieved success rates of 88.3% at six months, increasing to 97.6% at twelve months [28].

Treatment efficacy correlated significantly with cyst characteristics. Messaritakis (1991) showed that smaller hepatic cysts were more likely to be successfully treated (3.93 ± 1.87 cm vs. 7.78 ± 3.08 cm, *p* < 0.001), supporting size-based treatment guidelines [34].

Demirbilek (2001) documented the progressive ultrasonographic changes during medical therapy, following a predictable pattern: detachment of the endocyst, disappearance of regular endocyst, reduction in fluid component and cyst size, culminating in a solid appearance of the cyst remnant indicating complete cure [22]. The average time for the development of this solid appearance was 13 months (range 11–16 months), providing valuable predictive information for treatment duration expectations [22].

#### 3.3.3. Surgical Approaches and Techniques

Surgical management has evolved markedly, shifting from traditional open procedures to minimally invasive techniques (MIT). Size-based surgical selection criteria were consistently implemented, with thresholds ranging from 4 cm to 5 cm [28,31,32]. Azizoğlu (2024) documented that surgical intervention was primarily indicated for larger cysts (average size 100 ± 30.5 mm in surgical group versus 60 ± 30.8 mm in non-surgical group, *p* = 0.000) [6], providing statistical validation for size-based decision algorithms.

Comparative analyses by Masood (2022) and Minaev (2017) [26,27], reported notable advantages of MIT over open approaches, including shorter hospital stays (5.6 ± 2.2 versus 12.1 ± 1.5 days, *p* < 0.05), reduced operation time (90.1 ± 7.8 versus 120.6 ± 5.3 min, *p* < 0.01), and decreased analgesic requirements (1 day versus 3.1 ± 1.2 days, *p* < 0.01). Masood (2022) conducted a controlled study with 30 laparoscopic and 30 open procedures, finding comparable patient demographics and cyst characteristics between groups, strengthening the validity of their comparative findings [26].

Technical details of surgical approaches were documented across studies. The traditional open approach typically employed a right subcostal incision with field protection using cetrimide or betadine-soaked packs [26,35]. The laparoscopic technique utilized pneumoperitoneum (12 mmHg) with a 30-degree scope through a 5 mm umbilical port and placement of additional 5–10 mm ports [26]. Minaev (2017) detailed the use of ultrasonic Harmonic scalpels, bipolar coagulation (EnSeal), and argon coagulation of the exocyst wall during laparoscopic procedures, a critical step to prevent biliary fistulas and local recurrence [27].

Field protection techniques were universally employed, using betadine-soaked or waterproof towels to prevent spillage and secondary echinococcosis. Scolicidal agents varied in composition and concentration, including 10% hypertonic saline [32], 20% hypertonic saline [31], 3% hypertonic saline or cetrimide [26], and 10% formalin solution [35], with application durations ranging from 5 to 10 min. Wu (1992) specified that the aspirated volume of scolicidal agent never exceeded the evacuated fluid volume, providing a quantifiable safety parameter to prevent sclerosant-induced complications [35].

Management of the residual cavity emerged as a critical determinant of postoperative outcomes. Various techniques were documented, including omentoplasty (predominant approach in Masood, 2022 [26]; 65% of open cases in Minaev, 2017 [27], external tube drainage, capitonnage, marsupialization, and partial pericystectomy. Minaev (2017) reported employing omentoplasty in 65% of open cases, with marsupialization (28.3%) and tube drainage (6.7%) utilized for the remainder [27]. The laparoscopic approach incorporated an advanced technique where the omentum was secured within the residual cavity and fixed with separate stitches to the fibrous capsule edge.

Demirbilek (2001) evaluated various cavity management techniques, documenting that those complications were more frequent in the tube drainage group. Abscesses occurred in 27% of cases, cholangitis in 9%, and long-term biliary fistulas in 18% [22]. Cystectomy was associated with lower complication rates (12% cyst abscess, 6% cholangitis). Capitonnage resulted in 13% developed cyst abscess. Importantly, no early postoperative complications were observed with omentoplasty, suggesting superior outcomes with this technique [22].

Wu (1992) specifically compared open drainage versus capsulorrhaphy without drainage [35]. Ran (2015) classified surgical approaches as radical surgery (RS), including pericystectomy or segmentectomy and conservative surgery with partial pericystectomy (CSP) [30]. In their cohort of 112 pediatric patients, 26 underwent RS, and 86 received CSP, with partial cystectomy plus external drainage being the predominant approach (56 patients).

Despite theoretical advantages of definitive excision with radical approaches, Ran (2015) observed no significant differences in hospitalization duration or postoperative complications between radical and conservative techniques [30]. Operative times were longer for radical procedures (126.36 ± 36.97 versus 90.42 ± 22.89 min, *p* < 0.001), though without corresponding improvement in outcomes [30].

#### 3.3.4. Percutaneous Intervention Techniques

Percutaneous interventions, particularly PAIR, and catheterization techniques, are effective minimally invasive approaches for appropriately selected cases. Sağ (2022) utilized PAIR for cysts <6 cm with fluid content <100 cc (8 patients, 26.6%) and catheter-based methods for cysts >6 cm with content >100 cc (10 patients, 33.3%) [31].

Dolanbay (2020) compared PAIR with medical therapy in pediatric patients, showing distinct improvement patterns between groups (Dolanbay et al., 2020), although serological marker changes were not statistically significant [24].

Goktay (2005) provided extensive comparative data on percutaneous techniques in 34 patients with 51 hepatic hydatid cysts, comparing PAIR with catheterization. The technical success rate was 97.1%, with only one patient requiring surgical intervention for biliary fistula management. High intracystic pressure indicating cyst viability and endocyst separation from pericyst after hypertonic saline injection in 82.4% of patients during the initial 20-min waiting period, considered pathognomonic for viable hydatid cysts undergoing inactivation [25].

Follow-up data after percutaneous intervention demonstrated progressive cyst involution with mean volume reductions of 62.5% at 3 months, 66% at 6 months, 81.4% at 1 year, 85.1% at 2 years, and 88.2% final reduction, with no recurrence during the mean 3.1-year follow-up period [25].

Hospitalization times were shorter for percutaneous approaches compared to surgical interventions. Sağ (2022) documented that PAIR patients averaged 2.2 ± 0.4 days versus 4.5 ± 1.6 days for open liver procedures (*p* < 0.001), while catheter patients stayed 3.7 ± 0.9 days [31]. This reduction in hospitalization represents an important consideration in pediatric populations, where minimizing hospital stays offers substantial psychosocial benefits.

Complication profiles of percutaneous approaches were favorable. Pain was the most common post-procedural symptom (47.1%); however, only 8.8% required analgesic medication beyond 12 h. Minor complications included urticaria (8.8%) and fever (5.9%). Major complications occurred in 5.9% of catheterization patients [25]. These included one case of polymicrobial infection requiring catheter drainage and antibiotics, and another developing biliary communication necessitating surgical intervention.

#### 3.3.5. Complication Management

Biliary complications represented a significant consideration in hepatic hydatid disease management. The reported rates of biliary fistulas were 4.8% and 3.3% in laparoscopic and open cases, respectively [27]. Postoperative bile leakage occurred in 7% of patients [28]. Cysto-biliary communications (CBCs) were detected in 6.7% of laparoscopic and 3.3% of open procedures [26].

Standardized approaches for managing biliary leakage were established, with treatment decisions based on volume stratification. Oral (2012) reported that leakage volumes <100 mL/24 h (3.2% of patients) closed spontaneously after an average of 14 days, while volume leakage >100 mL/24 h (3.8% of patients) required endoscopic sphincterotomy via ERCP with subsequent closure after a mean of 33 days [28].

Technical innovations to prevent biliary complications included argon coagulation of the exocyst wall during laparoscopic procedures [27], and methylene blue injection through the cystic duct to detect connections (Ran et al., 2016). Despite these preventive measures, Ran et al. documented bile drainage in seven patients 2–4 days postoperatively, highlighting incomplete prevention [30].

Recurrence represented another challenge, ranging from 0 to 17.1% [32,35]. Multivariate analysis identified independent risk factors, including intimate contact of the cyst with large vessels (Odds ratio = 58.19, *p* = 0.011) and the presence of intraperitoneal effusion (Odds ratio = 65.5, *p* = 0.018) in Yosra’s (2023) cohort [32]. Non-compliance with prescribed medical therapy showed a significant correlation with recurrence (*p* = 0.010), emphasizing the importance of adherence to combined treatment approaches [32]. The summary of clinical outcomes is shown in Table 2.

#### 3.3.6. Treatment Algorithms and Patient Selection

Strategic patient selection and tailored treatment algorithms emerged consistently across studies. Cyst size represented the primary determinant in management decisions, with clearly defined thresholds guiding intervention selection. Small cysts (<5 cm) were primarily managed with medical therapy using albendazole [28,31]. Medium-sized cysts (5–6 cm) were treated with PAIR in combination with adjunctive albendazole for appropriate anatomical locations [31]. Large cysts (>6 cm) required catheter drainage or surgical intervention, depending on anatomical considerations.

Beyond size, additional factors influenced treatment selection. Cyst location and accessibility determined surgical approaches, with pericystectomy preferred for exophytic cysts and segmentectomy utilized for multiple left lateral segment cysts [30]. Multi-organ involvement necessitated staged treatment, where lung pathology was addressed first via thoracotomy, followed by liver interventions one month later [30,31]. Biliary involvement required modified management, incorporating endoscopic sphincterotomy and specialized drainage approaches [28]. Cyst complexity, classified according to the Gharbi/WHO-IWGE systems, also guided specific intervention selection [27,30].

**Figure 2:**
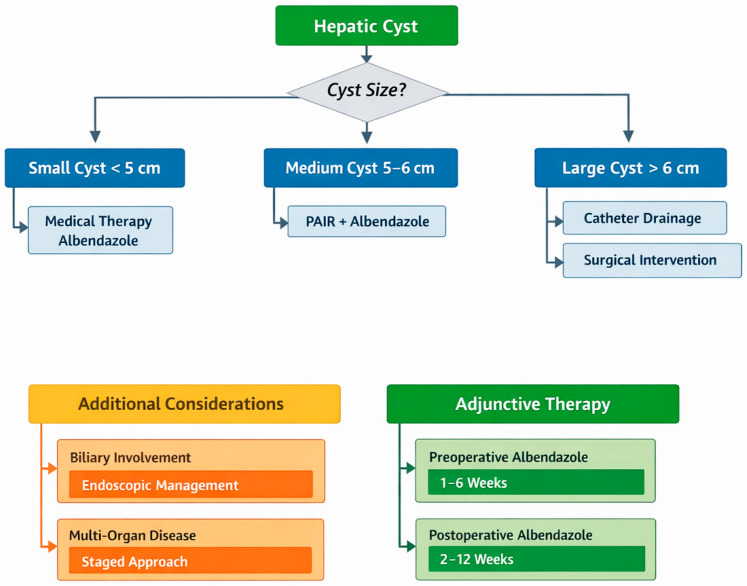
Treatment Algorithms and Patient Selection.

Combined therapy approaches emerged as standard practice, with albendazole administration before and after interventional procedures. Albendazole was commonly used perioperatively, although the protocols differed. Preoperative administration ranged from 1 to 6 weeks, with most studies implementing a 2–4-week course. Postoperative regimens demonstrated greater heterogeneity, ranging from 14 to 21 days [30] to extended three months [31] or cyclical treatment strategies [26,31] (Figure 2).

## 4. Discussion

### 4.1. Summary of Key Findings

This systematic review synthesized evidence from 15 studies on the management of pediatric hepatic hydatid disease, revealing several important patterns in diagnosis, treatment approaches, and outcomes. The diagnostic approach consistently employed multimodal strategies, with the US as the primary imaging modality supplemented by serological testing. Serological tests demonstrated variable sensitivity, with ELISA (84.4–86%) showing superior performance compared to IHA (60–66.1%). The clinical presentation patterns were consistent across studies, with abdominal pain representing the predominant symptom (55.4–77.1% of cases). Epidemiological factors, particularly residence in endemic areas and canine contact history were identified as significant risk factors, emphasizing the importance of preventive measures in these populations.

Our analysis of treatment modalities revealed a clear evolution in therapeutic approaches over time. Medical therapy protocols have standardized around albendazole (10–15 mg/kg/day), which has replaced mebendazole as the preferred pharmacological agent. The effectiveness of medical monotherapy was found to be significantly associated with cyst characteristics, particularly size. Small cysts (<5 cm) demonstrated success rates of 88.3–97.6% at 6–12 months. This finding substantiates the size-based treatment algorithms implemented across multiple studies.

Surgical management has evolved from traditional open approaches toward MIT. Comparative analyses between laparoscopic and open approaches demonstrated notable advantages for minimally invasive procedures, including shorter hospital stays (5.6 ± 2.2 versus 12.1 ± 1.5 days), reduced operation time (90.1 ± 7.8 versus 120.6 ± 5.3 min), and decreased analgesic requirements. Management of the residual cavity emerged as a critical determinant of postoperative outcomes. Omentoplasty demonstrated superior results compared to other techniques, such as tube drainage, which was associated with higher complication rates.

PAIR and catheterization techniques have emerged as effective approaches for appropriately selected cases. They demonstrated high success rates (97.1%) and favorable complication profiles. Hospitalization durations were shorter with percutaneous approaches compared to surgical interventions. This represents an important psychosocial benefit in pediatric populations.

The evolution toward more personalized treatment algorithms based on cyst size and location represents an important advancement in pediatric hydatid disease management [37]. These tailored approaches optimize outcomes as well as minimize procedural risks. This is crucial in pediatric populations where growth and developmental factors must be carefully balanced against treatment efficacy.

### 4.2. Previous Similar Studies

Our findings align with and extend previous research on hydatid disease management. Several prior meta-analyses have evaluated treatment outcomes in predominantly adult populations, but our study specifically addresses the unique considerations in pediatric cases. Sokouti et al. (2019) conducted a meta-analysis comparing PAIR and laparoscopic procedures for liver hydatid cysts in a broader population [13]. They reported that the PAIR approach had higher cure rates and lower complication and mortality rates than laparoscopic techniques, though laparoscopic procedures had a lower recurrence rate. Another meta-analysis by Sokouti et al. (2017) compared laparoscopic and open surgeries for liver hydatid cysts, finding no significant differences in postoperative complications, mortality, recurrence, or cure rates between the two surgical approaches [12].

Moreover, Velasco-Tirado et al. (2018) conducted a systematic review and meta-analysis encompassing a broader age range, which suggested that combining surgery or PAIR with benzimidazole therapy, such as albendazole, yielded better treatment outcomes than surgery alone. Additionally, their findings indicated that combined albendazole and praziquantel therapy resulted in higher scolicidal activity and improved cyst resolution compared to albendazole monotherapy [38]. While not exclusively focused on pediatric populations, these studies suggest that minimally invasive techniques like PAIR may offer advantages in terms of cure rates and safety profiles.

### 4.3. Theoretical Implications of Size-Based Treatment Selection

The consistent effectiveness of size-based treatment algorithms across multiple studies warrants further theoretical consideration. The differential response of hydatid cysts to various therapeutic modalities based on size may reflect fundamental biological characteristics of the parasite’s life cycle and host–parasite interactions [39]. Smaller cysts typically represent earlier stages of infection with potentially more permeable membranes, allowing better penetration of antiparasitic medications [40]. Conversely, larger cysts often develop more complex internal architecture with potential daughter cysts and calcifications that impede drug penetration [40].

This size-dependent response pattern suggests a critical therapeutic window wherein medical therapy alone may be sufficient, beyond which more invasive interventions become necessary [41]. The identification of this threshold (approximately 5 cm in most studies) provides a valuable clinical decision point that balances treatment efficacy with intervention-associated risks. This concept may have broader implications for parasitic disease management beyond hydatid disease, potentially informing treatment paradigms for other space-occupying parasitic infections such as neurocysticercosis, alveolar echinococcosis, cerebral paragonimiasis, and schistosomiasis with CNS involvement [42,43].

### 4.4. Interpretation and Implications

The findings of this systematic review have several important clinical and public health implications. First, the proven efficacy of medical therapy for small cysts supports a conservative first-line approach in carefully selected pediatric patients. This approach reduces procedural risks while preserving efficacy, a key consideration in pediatric patients where treatment must be balanced with growth and development.

Second, the superior outcomes of minimally invasive techniques compared to the traditional open approach suggest that these methods should be prioritized when intervention is necessary. While technical expertise and equipment availability may present barriers in resource-limited settings, the documented benefits in terms of reduced hospitalization, decreased analgesic requirements, and comparable efficacy make a compelling case for expanding access to these approaches.

Third, evidence supporting percutaneous interventions for medium-sized cysts offers an important intermediate option between medical therapy and surgery. This "middle ground" approach may be particularly valuable in pediatric populations.,. However, the implementation of these techniques requires specific expertise and equipment, highlighting the need for specialized centers with appropriate capabilities.

From a public health perspective, the documented relationship between canine contact history and disease prevalence underscores the continued importance of preventive measures in endemic regions [44]. Educational initiatives targeting hand hygiene, food safety, and responsible pet ownership represent critical complementary strategies to therapeutic interventions [45]. Additionally, the identification of treatment adherence as a key factor in preventing recurrence highlights the importance of patient education and structured follow-up.

Biliary complications, such as obstruction or fistula formation, remain a significant consideration in pediatric hepatic hydatid disease [46]. Endoscopic interventions, including sphincterotomy and specialized drainage techniques, have demonstrated efficacy in managing these complications and preventing recurrence [47]. Proactive identification of cysts involving the biliary tree and early intervention, combined with perioperative albendazole, represent key preventive strategies to minimize postoperative morbidity.

Barriers to implementation in low-resource or remote settings include limited access to specialized equipment, trained personnel, and dedicated pediatric centers [48]. Addressing these challenges will require capacity-building initiatives, telemedicine support, and targeted resource allocation to ensure equitable access to effective treatment modalities.

These findings collectively support a paradigm shift toward more personalized treatment algorithms based on cyst characteristics. It represents an important advancement in pediatric hydatid disease management, optimizing outcomes and minimizing procedural risks.

### 4.5. Strengths and Limitations

This systematic review has several notable strengths. First, our comprehensive search strategy across multiple databases yielded a diverse range of studies, including cohort studies, non-RCTs, and RCTs, providing a broad perspective on pediatric hydatid disease management. Second, our pediatric-specific focus addresses an important gap in the literature, as previous reviews have predominantly focused on adult populations or included mixed cohorts without separate analyses of pediatric outcomes. Third, our qualitative synthesis approach facilitated the integration of various study designs and outcome measures, allowing for a comprehensive evaluation of treatment modalities despite heterogeneity in reporting.

However, several limitations must be acknowledged. First, the heterogeneity in study designs, interventions, outcome measures, and patient characteristics prevent formal meta-analysis, limiting our ability to generate pooled effect estimates, and hinder direct comparisons. Second, the predominance of observational studies with small sample size and retrospective design introduces potential selection and reporting biases, with only one RCT included in our study. Third, variable follow-up periods across studies (ranging from months to several years) may impact the assessment of long-term outcomes, particularly recurrence rates.

Fourth, the inclusion of studies spanning several decades introduces potential confounding factors related to evolving diagnostic capabilities, treatment protocols, and supportive care measures. Fifth, the review excluded non-English studies, which may introduce language bias and limit the generalizability of findings, particularly from endemic regions where relevant studies may be published in local languages. Finally, while justified by its predominance, the focus on hepatic hydatid disease limits our findings’ applicability to other anatomical locations.

### 4.6. Conclusion and Recommendations

This systematic review evaluated the efficacy and safety of various treatment modalities for pediatric hepatic hydatid disease. Our findings support a personalized approach to management based primarily on cyst characteristics, particularly size. For small cysts (<5 cm), medical therapy with albendazole represents an effective first-line approach, with high success rates and favorable safety profiles. Medium-sized cysts (5–6 cm) can be managed with percutaneous interventions such as PAIR, particularly when combined with adjunctive albendazole therapy. Larger cysts (>6 cm) require more invasive management, with laparoscopic approaches demonstrating advantages over open surgery when technically feasible.

Based on these findings, we recommend the implementation of standardized treatment algorithms incorporating cyst size, location, and complexity as key decision points. We advocate for expanded access to minimally invasive techniques, including both laparoscopic surgery and percutaneous interventions, particularly in pediatric-focused centers. Continued efforts to enhance preventive measures in endemic regions remain essential, complementing therapeutic interventions with public health initiatives targeting primary prevention.

Future research should focus on prospective comparative studies with standardized outcome measures, particularly RCTs evaluating the long-term efficacy and safety of different treatment modalities in pediatric populations. Further, studies investigating the optimal duration and dosing of adjunctive medical therapy would address an important gap in current evidence.

## Figures and Tables

**Table 1:** Summary and baseline characteristics of the included studies.

ID	Study Design	Country	Sample Size	Intervention Details	Control Details	Diagnostic Tools	Follow-Up Period	Outcomes	Age	Sex (F/M)	Cyst Characteristics				Clinical Presentation	Lab Findings
											Number	Diameter	Sites in Liver	Isolated/Co-Existing		
Azizoğlu 2024	Retrospective cohort	Turkey	214	Operative + Albendazole (72): PAIR (28), Surgery (44): Laparoscopy (7), Laparotomy (37), Conversion to laparotomy (1)	Non-operative (142): Albendazole (108), Observation (34)	Ultrasound, CT, IHA, CBC, LFT	72 months	Cyst rupture, recurrence, cysto-biliary fistula, anaphylaxis, reoperation, mortality	11.1 ± 3.8	119/95	Single (145), Multiple (69)	<5 cm (72), 5–10 cm (97), >10 cm (45)	Right lobe (143), Left lobe (45), Bilateral (26)	184/30	Abdominal pain, mass in upper right quadrant, jaundice	Eosinophilia (55%), elevated bilirubin (37%), ALT (28%), AST (22%), GGT (17%), amylase (5%)
Yosra 2023	Retrospective descriptive and analytic study	Tunisia	122	Surgical removal of liver hydatid cysts (Albendazole was prescribed preoperatively in nine patients and postoperatively in 31 patients.)	-	US, serological tests	Median follow-up of 72 months	Cured rate, recurrence, complications	Median age 8.4 years (range 2.5–14 years)	49/73	190 cysts detected	Mean cyst size 6.6 cm	-	-	Abdominal pain, incidental findings	-
Masood 2022	Prospective randomized study	India	60	Laparoscopic surgery (All patients were treated with albendazole 15 mg/kg/d for at least 4 weeks preoperatively and continued postoperatively for a minimum 3 cycles with each cycle extending up to 3 weeks with 1-week gap)	Open surgery (All patients were treated with albendazole 15 mg/kg/d for at least 4 weeks preoperatively and continued postoperatively for a minimum 3 cycles with each cycle extending up to 3 weeks with 1-week gap)	US, CECT abdomen pelvis, IgG antibody	Minimum of 2 years	Recurrence, duration of hospital stay	Mean age 10.85 years (range 6–14 years)	27/33	Single (49), Multiple (11)	Laparoscopic group: Mean 8.8 cm (± 2.39 cm) Open group: Mean 8.97 cm (± 3.32 cm)	Laparoscopic group: Right lobe: 20 cysts Left lobe: 7 cysts Caudate lobe: 1 cyst Open group: Right lobe: 23 cysts Left lobe: 5 cysts Both lobes: 2 cysts	-	Symptoms varied based on organ involvement, common symptoms included abdominal pain and discomfort	-
Pradhan 2022	Retrospective cross-sectional study	India	14	Surgical excision and oral Albendazole was administered for at least 6 weeks during postoperative period to reduce the chances of recurrence.	-	US, CT, serological tests	None specified	Presentation	3 to 15 years (median 5.75 years)	4/10	11 hydatid cysts	Average 8.64 cm (liver), 6.1 cm (lung)	-	7/4	Right upper abdominal pain, cough, breathing difficulty, jaundice	-
Sağ 2022	Retrospective study	Turkey	35	Surgical interventions (thoracotomy, laparotomy, catheter, PAIR) (Albendazole (10 mg/kg/day) treatment was started on patients planned to undergo surgery three weeks before the operation).	-	US, CT	The patients were called to follow-up in postoperative 1st, 3rd, 6th, 9th and 12th months and every six months afterwards.	Recurrence, complications, duration of hospital stay	12 ± 3.13	15/20	-	73.2 ± 21.7 mm	Liver right lobe 22 (55.3%) Lung and liver 3 (7.9%) (Liver left lobe-lung left lower lobe; 2 liver right lobe-left lung lower lobe; liver left lobe-lung left upper lobe) (5.3%) Liver left lobe 1 (2.6%) Liver and kidney 1 (2.6%)	24/6 (Patients)	Abdominal pain, chest pain, cough	-
Aygün 2020	Retrospective review	Turkey	56	Medical treatment with albendazole; surgical treatment (cystotomy, capitonnage, PAIR), Surgical approach was considered for patients showing lung involvement.	-	Indirect hemagglutination test (IHA), radiological imaging (chest X-ray, abdominal/torax ultrasonography, CT, MRI).	None specified	Cured rate, complications	Median age 10.3 years (range 2.8–17.5 years).	26/30	Single (49), Multiple (7)	Median size: 6.0 cm (range 1–12 cm).	Right lobe of liver: 29 cysts Left lobe of liver: 15 cysts	24/17	Abdominal pain: 31 patients (55.4%), cough: 28 patients (50%), fever: 24 patients (42.9%). Other symptoms include chest pain, fatigue, hemoptysis, jaundice, skin rash, and asymptomatic cases.	Eosinophilia: Present in 17 patients (30.4%). Leukocytosis: Present in 21 patients (37.5%). Echinococcal IHA Positivity: 37 patients (69.1%).
Dolanbay 2020	Retrospective study	Turkey	27	Puncture Aspiration Injection Re-aspiration (PAIR) procedure	Patients who did not undergo PAIR	Serology, radiological imaging (ultrasound)	-	Cured rate, Improvement rate	11.59 ± 4.95	10/17	27 patients	-	-	-	Symptoms included abdominal pain, nausea, vomiting, respiratory distress, cough, headache, and impaired hearing/vision	All patients were seropositive for hydatid disease
Minaev 2017	Non-RCT	Russia	81	Laparoscopic surgery (Albendazole was administered before (1 course) and after (2–4 courses) surgery in all children (10 mg/kg per day, administered twice daily).	Open surgery (Albendazole was administered before (1 course) and after (2–4 courses) surgery in all children (10 mg/kg per day, administered twice daily).	US, CT	12 to 24 months	Complications, recovery time	9.3 ± 2.1	44/7	-	-	Right lobe: 90.4% in both groups	-	-	-
Ran 2015	Retrospective study	China	112	Radical surgery (All patients with CE were administered 10 mg/kg albendazole per day postoperatively for 14 to 21 days.)	Conservative surgery (All patients with CE were administered 10 mg/kg albendazole per day postoperatively for 14 to 21 days.)	US, CT	One year	Cured rate, recurrence, complications, duration of hospital stay	Average age 7.86 years (radical surgery group), 9.82 years (conservative surgery group)	46/66	194 hydatid cysts	Mean diameter 8.40 cm (radical surgery group), 8.81 cm (conservative surgery group)	Right lobe: 34 patients Left lobe: 15 patients Both lobes: 37 patients (conservative surgery group)	Co-infections with lung cysts in 22 patients	Abdominal pain, cough, expectoration, chest pain, abdominal mass	-
Oral 2012	Retrospective review	Turkey	156	Small liver cysts (<5 cm) were treated with medication alone. Surface liver cysts (>5 cm) required surgery plus medication, while deep parenchymal cysts (>5 cm) were managed with percutaneous drainage and medication. Treatment continued for six months post-therapy.		US, CT, IHA test	1 to 10 years (median 6.5 years)	Cured rate, Improvement rate, complications, and mortality	Average age 9.2 years (range 1.1–15 years)	64/92	376 cysts	Mean size: 6.82 cm (range 2–18 cm)	-	268/108	Abdominal pain: 122 patients, Abdominal mass: 92 patients, Fever: 86 patients, Weight loss: 42 patients, Asymptomatic: 31 patients, Jaundice: 12 patients, Acute abdomen: 5 patients, Urticaria: 5 patients.	Positive IHA test: Mean rate of 72% for liver hydatidosis
Goktay 2005	Non-RCT	Turkey	34	Ultrasound-guided percutaneous treatment with albendazole prophylaxis	-	US, serology	1 to 6 years (mean 3.1 years)	Cured rate, duration of hospital stay	4 to 17 years (mean 9.4 years)	19/15	51 hydatid liver cysts	3 to 16 cm (mean, 7.8 cm)	-	-	Right upper abdominal discomfort, pain, tenderness	Positive serology in 27 of 32 patients
Demirbilek et al. 2001	Retrospective cohort	Turkey	102	Albendazole (10 mg/kg b.i.d. p.o.) (67) Surgical procedures (cystectomy, tube drainage, capitonnage, omentoplasty) (35 primarily and 49 after unsuccessful medical treatment)	-	US, CT	14 months to 9 years (mean 32 months)	Cured rate, recurrence, complications, duration of hospital stay	4 to 15 years (mean 8.15 years)	38/64	Single (77), Multiple (25)	Over 5 cm: 87 cysts Less than 5 cm: 42 cysts	Right lobe: 100 cysts Left lobe: 45 cysts	84/18	-	Elevated liver function tests in 5 patients (excluded from medical therapy group)
Khursheed 2001	Non-RCT	India	42	Capitonnage (Postoperatively, all the patients received oral albendazole 10 mg/kg per day for three 28-day courses with a month rest period in between).	Open method (Postoperatively, all the patients received oral albendazole 10 mg/kg per day for three 28-day courses with a month rest period in between).	US, CT	Median 11 months (range 8 to 14 months)	Postoperative complications, recovery time	2 to 14 years	15/35	73 hydatid cysts	-	-	-	Epigastric discomfort, abdominal mass	-
Wu 1992	Non-RCT	China	43	Open drainage (All children were treated with oral mebendazole in doses of 50–100 mg/kg/day for 28–30 days after surgery.)	Capsulorrhaphy without drainage (All children were treated with oral mebendazole in doses of 50–100 mg/kg/day for 28–30 days after surgery.)	US, CT	Median follow-up of 42 months	Cure rate, complications, duration of hospital stays	2 to 14 years	14/15	35 cysts in open drainage group, 46 in capsulorrhaphy group	The removed cysts varied from 3 to 20 cm in diameter	-	-	Symptoms related to hepatic hydatid disease	-
Messaritakis 1991	Prospective study	Greece	39	Mebendazole (100–200 mg/kg/day for 12 weeks)	None specified	Radiography, CT, US	Mean (SD) of 63 (24) months	Cured rate	2 to 14 years	22/17	Liver: 37 cysts	-	-	-	-	-

**Table 2:** Summary of the outcomes of the included studies.

ID	Arms	Total	Outcomes					
			Cured	Improvement	Recurrence	Complications	Duration of Hospital Stay, Days, M ± SD	All-Cause Mortality
**Azizoğlu 2024**	Surgical + Albendazole	72	-	-	2	16	-	0
	Non-operative: Medical treatment (Albendazole) OR Observation without treatment	142	-	-	2	0	-	0
**Yosra 2023**	Surgical (Albendazole was prescribed preoperatively in nine patients and postoperatively in 31 patients.)	122	-	-	20	29	13.9 ± 10.8	-
**Masood 2022**	Laparoscopic surgery plus albendazole	30	-	-	0	2	4.57 ± 1.33	0
	open surgery plus albendazole	30	-	-	0	4	7.07 ± 1.89	0
**Pradhan 2022**	Surgical excision plus albendazole	11	-	-	0	-	-	-
**Sağ 2022**	Surgical	12	-	-	5	3	2.2 ± 0.4	-
	Catheterization	10	-	-		-	3.7 ± 0.9	-
	PAIR	8	-	-		-		-
**Aygün 2020**	Surgery and medical	21	21	21	0	-	-	0
	Only medical	15	15	15	0	1	-	0
	PAIR and medical	5	5	5	0	-	-	0
**Dolanbay 2020**	PAIR	10	-	10	-	-	-	-
	Non-PAIR	17	-	17	-	-	-	-
**Minaev 2017**	Laparoscopic surgery plus albendazole	21	-	-	0	3	5.6 ± 2.2	-
	Open surgery plus albendazole	60	-	-	0	11	12.1 ± 1.5	-
**Ran 2015**	Radical surgery + Albendazole	26	-	26	0	5	11.00 ± 2.14	-
	Conservative surgery + Albendazole	86	-	83	3	13	11.46 ± 3.12	-
**Oral 2012**	Surgical + Albendazole	113	72	20	10	11	-	0
	Albendazole	43	30	8	5	-	-	0
**Goktay 2005**	US–guided percutaneous treatment with albendazole	13	-	-	0	1		0
	Catheterization	27	-	-	0	0		0
	Combination	6	-	-	0	0		0
**Demirbilek 2001**	Surgical	84	-	-	2	12	8.57 ± 5.77	-
	Albendazole only	67	-	18	1	-	-	-
**Khursheed 2001**	Capitonnage	23	-	-	-	6	12.96 ± 2.3	0
	Open method	19	-	-	-	2	10.05 ± 1.64	0
**Wu 1992**	Open method plus mebendazole	22	-	-	0	0	Mean 8.5	0
	Capsulorrhaphy plus mebendazole	21	-	-	-	0		0
**Messaritakis 1991**	Mebendazole	39	20	-	-	11	-	0

## Data Availability

No new data were created or analyzed in this study.

## Supplementary Materials

The following supporting information can be downloaded at: https://www.mdpi.com/article/10.3390/pediatric18020038/s1, File S1: PRISMA 2020 Checklist [18].

## Abbreviations

The following abbreviations are used in this manuscript:

PAIR	Puncture, Aspiration, Injection, and Re-aspiration
PRISMA	Preferred Reporting Items for Systematic Reviews and Meta-Analyses
WoS	Web of Science
MeSH	Medical Subject Headings
NOS	Newcastle–Ottawa Scale
RCT	Randomized Controlled Trial
ROBINS-I	Risk of Bias in Non-Randomized Studies of Interventions
ROB-2	Risk of Bias 2 tool
US	Ultrasound
CT	Computed Tomography
IHA	Indirect Hemagglutination Assay
ELISA	Enzyme-Linked Immunosorbent Assay
MIT	Minimally Invasive Techniques
RS	Radical Surgery
CSP	Conservative Surgery with Partial Pericystectomy
CBCs	Cysto-Biliary Communications
ERCP	Endoscopic Retrograde Cholangiopancreatography
WHO-IWGE	World Health Organization–Informal Working Group on Echinococcosis
CNS	Central Nervous System

## Appendix A

**Table A1:** Quality Assessment of Cohort Studies Using the Newcastle–Ottawa Scale (NOS). Number of asterisks equals the number of NOS criteria met and that more asterisks mean higher methodological quality according to the NOS.

Study ID	Selection	Comparability	Outcome	Overall	Quality
**Azizoğlu 2024**	****		**	6	Fair
**Yosra 2023**	****	**	***	9	Good
**Sağ 2022**	***	*	***	7	Good
**Aygün 2020**	***	*	**	6	Fair
**Dolanbay 2020**	***	*	**	6	Fair
**Ran 2015**	***	**	**	7	Good
**Oral 2012**	****	**	***	9	Good
**Demirbilek 2001**	****		***	7	Good

## Appendix B

**Table A2:** Quality Assessment of Non-Randomized Controlled Trials (Non-RCTs) Using the Risk of Bias in Non-randomized Studies of Interventions (ROBINS) Tool.

ID	Confounding	Selection	Classification	Deviations	Missing Data	Measurement	Reporting	Overall Risk
**Minaev et al. (2017)**	Low	Low	Low	Low	Low	Low	Low	Low
**Goktay et al. (2005)**	Moderate	Low	Low	Low	Low	Low	Moderate	Moderate
**Khursheed et al. (2001)**	Serious	Moderate	Low	Low	Moderate	Moderate	Serious	Serious
**Wu et al. (1992)**	Moderate	Moderate	Low	Low	Moderate	Moderate	Serious	Moderate to Serious
**Messaritakis et al. (1991)**	Moderate	Low	Low	Low	Moderate	Low	Moderate	Moderate

## Appendix C

**Table A3:** Quality Assessment of a Cross-Sectional Study Using the Newcastle–Ottawa Scale (NOS). Number of asterisks equals the number of NOS criteria met and that more asterisks mean higher methodological quality according to the NOS.

ID	Selection	Comparability	Outcome	Overall	Quality
**Pradhan 2022**	****		**	6	Fair

## Appendix D

**Table A4:** Quality Assessment of a Randomized Controlled Trial (RCT) Using the Risk of Bias 2 (ROB-2) Tool.

ID	D1	D2	D3	D4	D5	Overall
**Masood 2022**	Some concerns	Low	Low	Low	Some concerns	Some concerns

D1: Bias arising from the randomization process. D2: Bias due to deviations from intended intervention. D3: Bias due to missing outcome data. D4: Bias in measurement of the outcome. D5: Bias in selection of the reported result.
